# Inactivation of Respiratory Syncytial Virus by Ozone Generated via Dielectric Barrier Discharge Technology with Decrease in Intact Viral Surface Protein

**DOI:** 10.3390/microorganisms13112611

**Published:** 2025-11-16

**Authors:** Akikazu Sakudo, Ryoya Moriyama, Masanori Nieda

**Affiliations:** 1 School of Veterinary Medicine, Okayama University of Science, Imabari 794-8555, Ehime, Japan; 2 OHNIT Co., Ltd., Akaiwa 709-0802, Okayama, Japan

**Keywords:** DBD, dielectric barrier discharge, disinfection, inactivation, ozone, plasma, RSV

## Abstract

Respiratory syncytial virus (RSV) is a major cause of severe respiratory infections, particularly in infants and young children. Although disinfection methods using alcohol and detergents are effective, their application in pediatric environments poses safety concerns. Ozone (O_3_) has been employed for water treatment, food preservation, and air purification, but its efficacy against RSV has not been well studied. Here, we investigated the inactivation of RSV using a dielectric barrier discharge (DBD)-based ozone generator (SFG1210). The RSV A2 strain was spotted on glass coverslips and exposed to low-concentration ozone (0.5 ppm) for 1 h under controlled temperature (24.6~27.2 °C) and relative humidity (71.9~75.1%) conditions. Subsequent infectivity assays combined with immunochromatography showed that ozone exposure significantly reduced RSV infectivity. Specifically, viral titration assay of median tissue culture infectious dose (TCID_50_) showed that RSV titers were reduced by more than 6 logs. In addition, biochemical analyses showed significant reductions in intact RSV genomic RNA and F protein levels after ozone treatment, suggesting that ozone inactivates RSV by damaging both the viral genome and surface proteins. These findings demonstrate the potential applicability of the SFG1210 ozone generator as an effective tool for surface disinfection of RSV, providing a safe, non-contact, and practical approach for infection control in healthcare and childcare settings.

## 1. Introduction

Respiratory viruses, including influenza virus, respiratory syncytial virus (RSV), and coronaviruses, pose significant threats to global public health due to their high transmissibility and potential to cause severe disease outbreaks [1]. The recent coronavirus disease 2019 (COVID-19) pandemic caused by severe acute respiratory syndrome coronavirus 2 (SARS-CoV-2) has highlighted the urgent need for effective environmental disinfection methods that can mitigate viral spread not only through direct person-to-person contact but also via contaminated surfaces [2,3].

RSV is a major cause of severe lower respiratory tract infections, particularly in infants, young children, and immunocompromised individuals [4]. The virus is primarily transmitted through respiratory droplets generated by coughing and sneezing (droplet transmission), although indirect contact with contaminated surfaces or objects also constitutes a major route of transmission [5]. Consequently, surfaces can become contaminated and serve as sources of infection, particularly in places frequented by children such as early childhood education and pediatric daycare centers [6].

The inactivation methods of RSV, an enveloped virus, utilize physical or chemical approaches. Several studies show that thermal treatment at ≥50 °C rapidly reduces viral infectivity. For example, a 1969 study reported an 11-log reduction in RSV infectivity per hour at 50 °C [7]. Some RSV strains (RSV/Sendai/851/13) are completely inactivated after 24 h incubation at 4 °C [8]. Cold oxygen plasma (COP) alone and COP + ultraviolet-C (UV-C) exposure achieved 2.0-log and 3.8-log reductions in RSV infectious titer, respectively [9]. Both cold oxygen plasma (COP) and ultraviolet (UV) exposure generate ozone. Thus, ozone may, at least in part, contribute to the inactivation mechanisms of RSV [9].

Chemical approaches also result in a rapid and substantial reduction in RSV virucidal activity [10]. For example, alcohols such as 70% isopropanol [11,12] and ethanol [13] effectively inactivate RSV. Chlorine-based disinfectants (using sodium hypochlorite at 0.525% to 0.0525%) yield > 3-log reductions after exposure for 5 min at 4 °C [13]. Other agents such as fatty alcohols and lipids also inactivate both laboratory strains and clinical isolates of RSV through disruption of the viral envelope, although efficacy is influenced by factors such as pH and organic load (i.e., addition of milk or fruit juice) [12]. Moreover, reagents such as alcohol and detergents present a considerable risk of accidental ingestion for infants and young children. Therefore, the use of such reagents should be restricted in infection control practices. As such, there is an urgent requirement to develop safe and effective strategies for reducing the risk of RSV infection in environments where infants are present.

Ozone disinfection has emerged as a promising alternative strategy for the control of respiratory viruses, but its effectiveness is highly dependent on several parameters such as ozone concentration, exposure time, and relative humidity (RH) [14,15]. High levels of RH enhance ozone–water interactions that generate hydroxyl radicals (·OH), thereby increasing virucidal activity while enhancing ozone-induced degradation of materials [16,17]. Indeed, findings from other studies suggest that ozone inactivation of viruses is humidity-dependent. For example, maximal disinfection effects were observed at 70% RH for SARS-CoV-2 [18] and 80% RH for bacteriophage PhiX174 [19]. A recent study found the inactivation rate of viruses increased with elevated temperature (more than 20 °C) for influenza virus and higher RH (about 50~85%) for both influenza virus and corona virus [20]. Thus, temperatures of 20–35 °C and high RH (about 50~85%) appear to enhance ozone induced inactivation of viruses, most likely by promoting radical formation and facilitating oxidative reactions on viral surfaces.

A growing body of research has demonstrated that ozone can effectively inactivate respiratory viruses. However, there are no published studies reporting RSV inactivation mediated by ozone. A preprint from Blanchard and colleagues demonstrates that RSV on personal protective equipment exposed to 20 ppm ozone at an RH above 70% for 40 min or longer results in >99.99% reduction in viral infectivity [17]. This inactivation effect was also observed for influenza A virus [17]. In another study, Dubuis and co-workers reported that airborne influenza A virus underwent a 4-log reduction when exposed to approximately 1.7 ppm ozone at 76% RH for 80 min [16]. Hudson et al. [21] tested 12 viruses, including influenza virus (H3N2), herpes simplex virus (HSV), rhinovirus types 1A and 14, adenovirus types 3 and 11, mouse coronavirus (MCV), Sindbis virus (SINV), yellow fever virus (YFV), vesicular stomatitis virus (VSV), poliovirus (PV), and vaccinia virus (VV). Ozone gas at 20-25 ppm combined with >90% RH inactivated all of the tested viruses on surfaces by at least 3 log. Together, these findings show that viruses can be inactivated by ozone and highlight the importance of humidity in this process. Given the limitations of conventional disinfection methods (i.e., concerns related to toxicity and general applicability), there has been growing interest in the use of ozone as a possible disinfectant. Ozone has the ability to inactivate a wide range of pathogens, including viruses, and is compatible with most materials leaving behind no residue [18,22,23,24,25,26,27].

Ozone is a triatomic molecule (O_3_) known for its high oxidative potential. According to the Handbook of Chemistry and Physics [28], the standard electrode potential of ozone is +2.07 V. Ozone has long been used in water treatment, food preservation, and air purification [29,30]. More recently, the virucidal activity of ozone against enveloped respiratory viruses has attracted considerable interest [16]. However, there are no published reports regarding the inactivation activity of ozone against RSV, although one such study is currently in preprint [17].

The SFG1210 ozone generator utilizes a dielectric barrier discharge (DBD) configuration, where the electrodes are fully embedded within a quartz glass dielectric layer [31,32]. By embedding the electrodes within the dielectric barrier, the SFG1210 overcomes the limitations of conventional discharge-based ozone generators. This type of fabrication yields superior resistance to variations in humidity, enhanced micro-discharge stability, improved durability, and reduced maintenance requirements [31,32]. In addition, unlike conventional DBD units with metal plates directly adjacent to the discharge gap, the electrodes in the SFG1210 are fabricated from conductive paste and sintered into the dielectric substrate. Thus, no metallic surfaces are exposed to the discharge space in SFG1210, which enhances the long-term performance and robustness of the device. Although there are several advantages to using SFG1210, inactivation performance of SFG1210 against viral pathogens, especially RSV, is unknown.

Here, an evaluation of the efficacy of the SFG1210 ozone generator by DBD from air for inactivating RSV was performed. In addition, associated changes in viral structural components following exposure to ozone were investigated.

## 2. Materials and Methods

### 2.1. RSV

RSV A2 strain (VR-1540; A subtype laboratory virus) (American Type Culture Collection (ATCC), Manassas, VA, USA) was used in the present study. HEp-2 cells (ATCC, CCL-23) were cultured in minimal essential medium (MEM) (Nacalai Tesque, Kyoto, Japan) supplemented with 10% fetal calf serum (FCS) at 37 °C in a 5% CO_2_ atmosphere.

### 2.2. Configuration of the Ozone Generator

**Figure 1:**
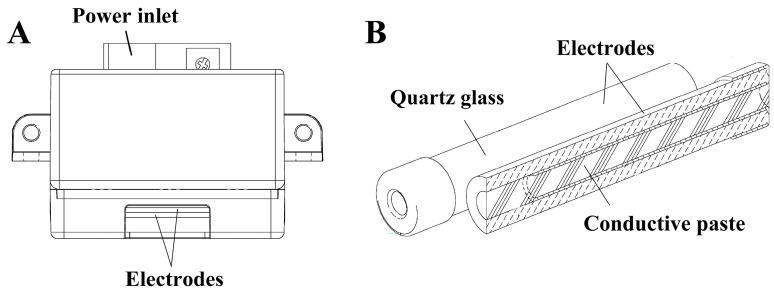
SFG1210 ozone generator. (**A**) SFG1210 ozone generator produces ozone by dielectric barrier discharge (DBD) derived from air. A power inlet and two electrodes are indicated. (**B**) The electrodes covered with a quartz glass dielectric layer as well as conductive paste are highlighted. Adapted with permission from OHNIT Co., Ltd. [31,32] and modified from Japanese Patent JP 5405296 B2 [33].

An SFG1210 ozone generator (SFG1210K-F, lot 21-079036, OHNIT Co., Ltd., Akaiwa, Japan) was used in this study [31,32]. The ozone generator incorporated conductive paste electrodes completely encapsulated in ceramic glass dielectric material (quartz glass) with a 0.3 mm discharge gap between the two electrodes (Figure 1). The power supply was 12 V direct current (minimum/maximum 10.7 V/13.6 V, respectively) and 114 mA (minimum/maximum 50 mA/230 mA, respectively). The SFG1210 device facilitated low-level stable production of ozone under varying conditions of ambient humidity and temperature [33].

### 2.3. Ozone Monitoring and Treatment

To assess the effect of ozone generated using SFG1210K-F, RSV samples were subjected to ozone treatment as follows. First, an ozone monitor (Ozone Monitor EG-3000, Ebara Jitsugyo Co., Ltd., Tokyo, Japan; detectable range: 0–1 ppm) was switched on 3 h before each experiment to ensure stability of readings. RSV samples (VR-1540, ATCC) were spotted (20 µL per spot) onto UV (ultraviolet)-sterilized glass coverslips (24 × 60 mm, thickness 0.12~0.17 mm; Matsunami Glass Ind., Ltd., Osaka, Japan). The samples were placed in a transparent box (desiccator: 500 × 345 × 335 mm; inner dimensions: 485 × 275 × 285 mm; AS ONE catalog no. 1-007-01) and exposed to 0.5 ppm ozone gas generated by the ozone generator for either 1 h or 24 h. To maintain uniform ozone distribution, a fan (model 07530GA-12L-AA; MinebeaMitsumi Inc., Nagano, Japan) was installed inside the box. A data logger (midi LOGGER GL240; Graphtec Corp., Tokyo, Japan) continuously monitored and regulated the concentration of ozone. Previous studies have shown that an RH of above 50% is required for optimal reactivity of ozone [16,17,34]. To maintain an appropriate RH, a 100 mm cell culture dish containing a saturated salt solution was placed inside the chamber. The saturated salt solution is a simple and reliable way to control and maintain a constant RH inside a closed chamber. In addition, RH and temperature were monitored using a thermo recorder (TR72wb-S; T&D Corp., Nagano, Japan). Nitrogen oxides (NOx) potentially generated alongside ozone were measured using an NOx detector (SAFEGAS Mini Meta-NOx; MK Scientific, Inc., Yokohama, Japan), which had a detection limit of 0.1 ppm.

The CT value, representing the product of ozone concentration (*C*, in ppm) and the treatment time (*t*, in min), was calculated as follows:

CT = *C* × *t*

As a control, the same setup was used with the fan operating but without ozone generation.

### 2.4. Detection of RSV Antigens

To detect RSV antigens, the Bionax NOW^®^ RSV test (Eiken Chemical Co., Ltd., Tokyo, Japan) and enzyme-linked immunosorbent assay (ELISA) (RSV-F ELISA Kit, KIT11049; Sino Biological, Inc., Kanagawa, Japan) were used according to the manufacturer’s instructions. The immunochromatography kit [35,36,37] and ELISA kit [38,39,40] specifically recognize RSV F protein.

### 2.5. Virus Recovery

After treatment with ozone and control counterparts, samples were recovered (i.e., collected from the treated spots by rinsing with SCDLP medium) using SCDLP (Soybean-Casein Digest Broth with Lecithin & Polysorbate 80) medium (SCDLP medium DAIGO; Shiotani M.S. Co., Ltd., Amagasaki, Japan). The recovered samples were subjected to the following assays.

### 2.6. Viral Titration Assay

For infectivity assays, HEp-2 cells were incubated for 24 h prior to infection. HEp-2 cells were then exposed to the treated samples diluted by 10-fold serial dilutions of samples in 2% FCS (fetal calf serum)-MEM (minimum essential medium) [41]. Samples were incubated at 37 °C in 5% CO_2_ for 5 days and subsequently analyzed using the Bionax NOW^®^ RSV test (Abbott, Abbott Park, IL, USA). In addition, to quantify infectious virus titers, TCID_50_ (median tissue culture infectious dose) assays using HEp-2 cells were conducted according to the Reed and Muench method [42]. The TCID_50_ assay was performed in a 100 µL volume per well.

### 2.7. Viral RNA Extraction and Real-Time Polymerase Chain Reaction (PCR)

To investigate whether genomic viral RNA is damaged by ozone treatment, viral genomic RNA was extracted and analyzed by real-time PCR. First, RSV RNA in ozone-treated and untreated samples was extracted using the QIAamp Viral RNA mini kit (Qiagen, Hilden, Germany) and solubilized in lysis buffer [37]. The extracted viral RNA was bound to a column and subsequently eluted following the manufacturer’s instructions. After incubation of the eluted RNA at 65 °C for 5 min, RNA was reverse-transcribed with random primers at 42 °C for 60 min, followed by denaturation of the enzyme at 95 °C for 5 min using a PrimeScriptII 1st strand cDNA Synthesis kit (Takara Bio Inc., Otsu, Japan) to prepare cDNA. Next, the intact viral genome was quantified using real-time PCR targeting the F protein gene of RSV using a SYBR premix Ex Taq II kit (Takara Bio Inc.) according to the manufacturer’s instructions.

The following PCR oligonucleotide primers specific for the RSV F gene [35,37] were used:

RSVfusion-F: 5′-TTAACCAGCAAAGTGTAAGA-3′

RSVfusion-R: 5′-TTTGTTATAGGCATATCATTG-3′

The temperature cycling program used in the QuantStudio 5 real-time PCR system (Applied Biosystems, Waltham, MA, USA) was as follows: denaturation (95 °C for 30 s), 40 cycles of denaturation (95 °C for 5 s), followed by annealing/extension (60 °C for 30 s). Each amplification reaction was performed in triplicate. The results were analyzed using QuantStudio Design & Analysis software v1.2 (Applied Biosystems). PCR specificity was verified by dissociation curve analysis of the amplified DNA fragments. In addition, the PCR product was sized by agarose gel electrophoresis. The anticipated sequence was validated by direct sequencing using an Applied Biosystems Big Dye Terminator V3.1 kit (Applied Biosystems, Waltham, MA, USA). Samples were analyzed on an Applied Biosystems 3730xl DNA Analyzer (Applied Biosystems).

### 2.8. Statistical Analyses

Statistical analyses were performed by the Mann–Whitney U test. A *p* < 0.05 was considered statistically significant using GraphPad Prism v7.02 software (GraphPad Prism Software Inc., La Jolla, CA, USA). Data are shown as the average ± SEM (standard error of the mean).

## 3. Results and Discussion

To accurately evaluate the antiviral efficacy of ozone generated by the SFG1210 device against RSV, it was essential to characterize the conditions in the sample box during operation. Importantly, it should be noted that viral inactivation by ozone is influenced by environmental parameters such as ozone concentration, exposure time, temperature, and RH. Therefore, these factors should be continuously monitored. Furthermore, ozone concentration profiles allowed the determination of the CT value. Visual inspection of ozone-treated viral spots provided preliminary evidence of oxidative effects by ozone.

**Figure 2:**
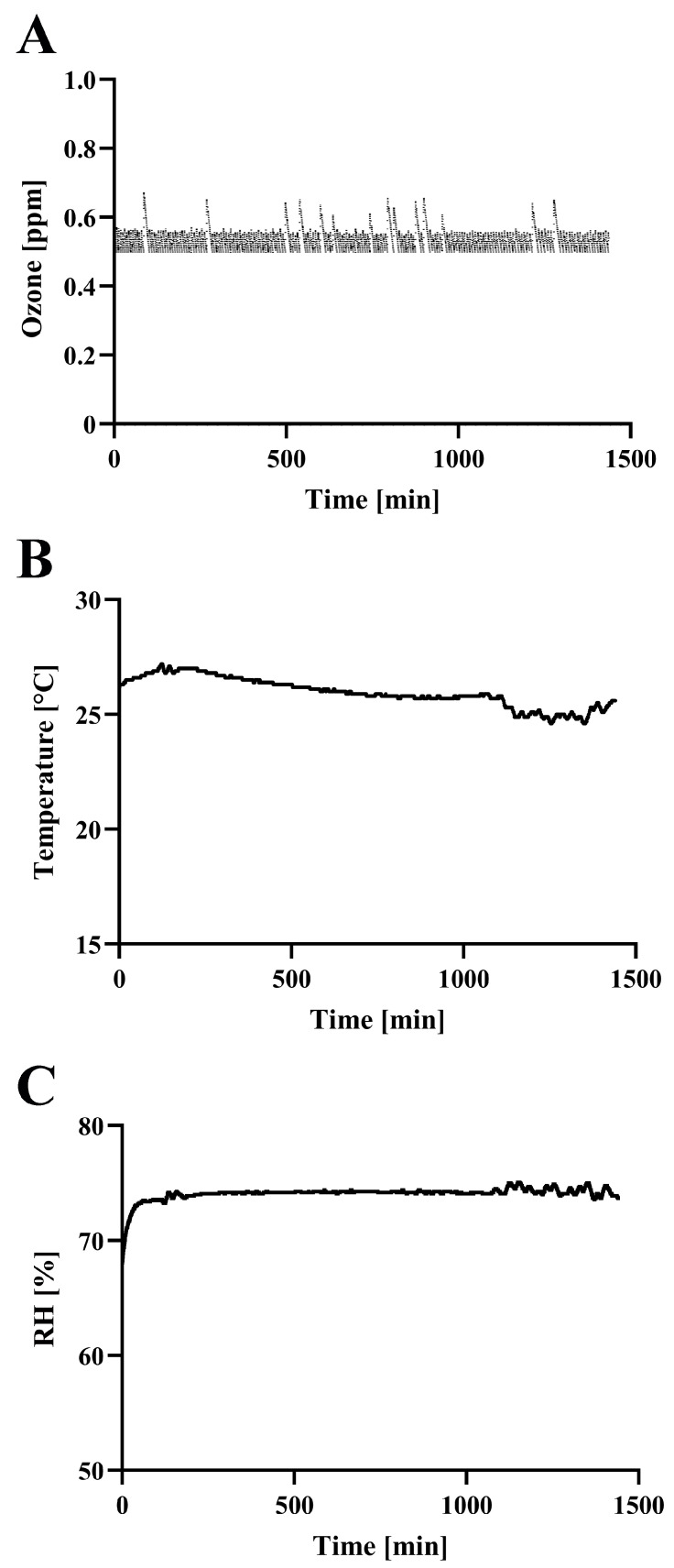
Ozone concentration, temperature, and relative humidity (RH) during treatment. The representative data of ozone concentration (**A**), temperature (**B**), and RH (**C**) measured by the methods described in the Section 2 during treatment are shown.

On the basis of the background considerations outlined above, we performed the following analysis. Initially, the concentration of ozone was measured along with the temperature and RH during operation of the SFG1210 ozone generator (Figure 2). The ozone concentration in the test box (0.038 m^3^; 485 × 275 × 285 mm) of SFG1210 reached 0.50 ppm within 160 s. Furthermore, the concentration of ozone was successfully controlled at around 0.5 ppm with several spikes (maximum concentration 0.674 ppm). These spikes in ozone concentration were likely due to calibration control interaction between the ozone generator and the ozone monitor. The generator was set to switch on at below 0.5 ppm and switch off at above 0.5 ppm, while the monitor performed automatic calibration every 5 min for about 20 s. During the calibration time, switch control was suspended. If the generator was turned on just prior to calibration, ozone would continue to be produced without feedback, leading to a transient increase in ozone levels. Once calibration was finished the generator switched off. This sequence of events explains the observed spikes in ozone concentration.

Next, CT values were calculated as described in Section 2. The representative CT value was 30.86 ppm·min at 1 h and 768.70 ppm·min at 24 h. Because the inactivation efficiency of ozone is affected by temperature and RH [20], these two factors were also monitored. Temperature and RH were well-controlled within 24.6~27.2 °C and 71.9~75.1%, respectively.

Moreover, NOx concentrations were measured. NOx are generated from air by high-energy electrons produced in the DBD. Specifically, the discharge excites and dissociates molecular nitrogen (N_2_) and oxygen (O_2_) present in the air. In this study, NOx concentrations measured at various time points (0, 5, 15, 30 min, 1, 2, 4, 6, 12 and 24 h) remained below the detection limit (<0.1 ppm). In addition, visual inspection of RSV spots revealed that 24 h ozone exposure (0.5 ppm) led to notable discoloration on the coverslip, changing from red to white (Figure S1), while 1 h exposure showed minimal color change (Figure S2). Therefore, we decided to use 1 h exposure of ozone in all further experiments, because the 24 h exposure caused marked discoloration of the spots, possibly due to oxidation and degradation of phenol-red in the medium. By contrast, the 1 h exposure minimized the influence of discoloration on the spots.

**Figure 3:**
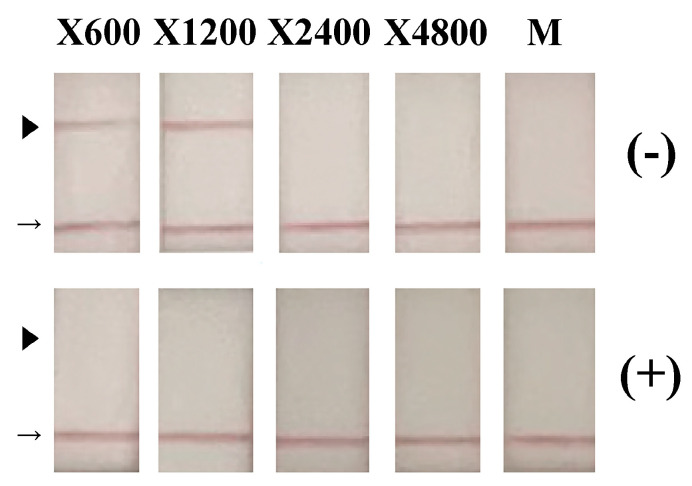
Inhibition of respiratory syncytial virus (RSV) proliferation in the HEp-2 cell culture after inoculation with ozone-treated virus and incubation. RSV (VR-1540) was exposed to 0.5 ppm ozone for 1 h (+) or to fan operation only (−). The treated samples were diluted in 2% FCS (fetal calf serum)–MEM (Minimum Essential Medium) and inoculated onto HEp-2 cells. After 5 days of incubation, the medium was collected and analyzed using an immunochromatographic assay targeting RSV F protein. Samples are arranged from left to right in order of increasing dilution: X600, X1200, X2400, X4800, and medium only (M). The arrowhead is the test line for the RSV F antigen, while the arrow is the control line, which is included in the kit as a positive control.

To investigate the inactivation activity of ozone generated by SFG1210 against RSV, viral samples were subjected to ozone treatment (0.5 ppm) for 1 h. Aliquots of cell culture medium containing RSV-infected HEp-2 cells were spotted onto glass coverslips (20 μL/coverslip) and subjected to ozone treatment. Samples on the coverslip were subsequently recovered and analyzed using a variety of biochemical methods. First, the recovered samples from the coverslips were diluted with culture medium and incubated with HEp-2 cells for 5 days. The recovered culture medium of HEp-2 cells incubated with ozone-treated VR-1540 virus showed no detectable RSV by immunochromatography even at 600-fold dilution (X600). By contrast, untreated control RSV yielded positive results even at 1200-fold dilution (X1200) (Figure 3). Infectivity of the recovered samples from the coverslips were assessed using a viral titration assay. TCID_50_ titers were 4.64 × 10^2^ TCID_50_/mL for ozone-exposed RSV and 6.11 × 10^8^ TCID_50_/mL for untreated RSV. Thus, 1 h exposure of RSV to 0.5 ppm ozone resulted in more than a 6-log reduction in infectivity.

**Figure 4:**
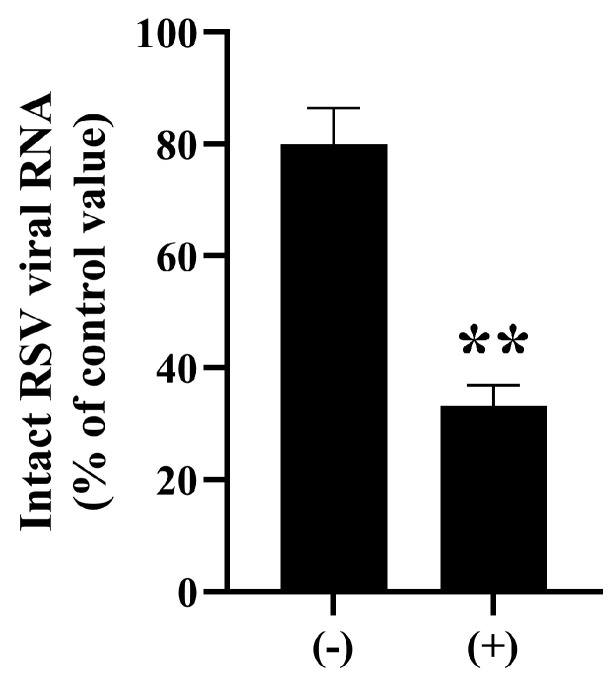
Decrease in intact RSV genomic RNA following ozone treatment. RSV (VR-1540) samples were treated with ozone (0.5 ppm, 1 h; (+)) or subjected to fan operation only (−). Quantification using real-time polymerase chain reaction (PCR) revealed a significant decrease in intact RSV genomic RNA in the ozone-treated group, indicating oxidative damage to viral RNA. Differences where *p* < 0.01 (**) versus control (−) were considered significant when verified by the Mann–Whitney U test.

Next, we investigated the effect of ozone treatment (0.5 ppm, 1 h) on the viral components. Real-time PCR analysis showed decreased levels of intact RSV genomic RNA in viral samples treated with ozone for 1 h in comparison to untreated control samples (Figure 4). Specificity of the real-time PCR was confirmed by dissociation curve analyses of the reaction products. Furthermore, quantitative real-time PCR analysis revealed that ozone treatment (33.19 ± 3.72%) significantly reduced intact genomic RNA levels to less than half compared to untreated controls (79.96 ± 6.49%) (Figure 4). Sequencing of the PCR amplicons (*N* = 3) confirmed 97~99% identity with 1~2 base gaps to the targeted gene (GenBank: MW582527.1 RSV A2 fusion protein gene, complete cds).

**Figure 5:**
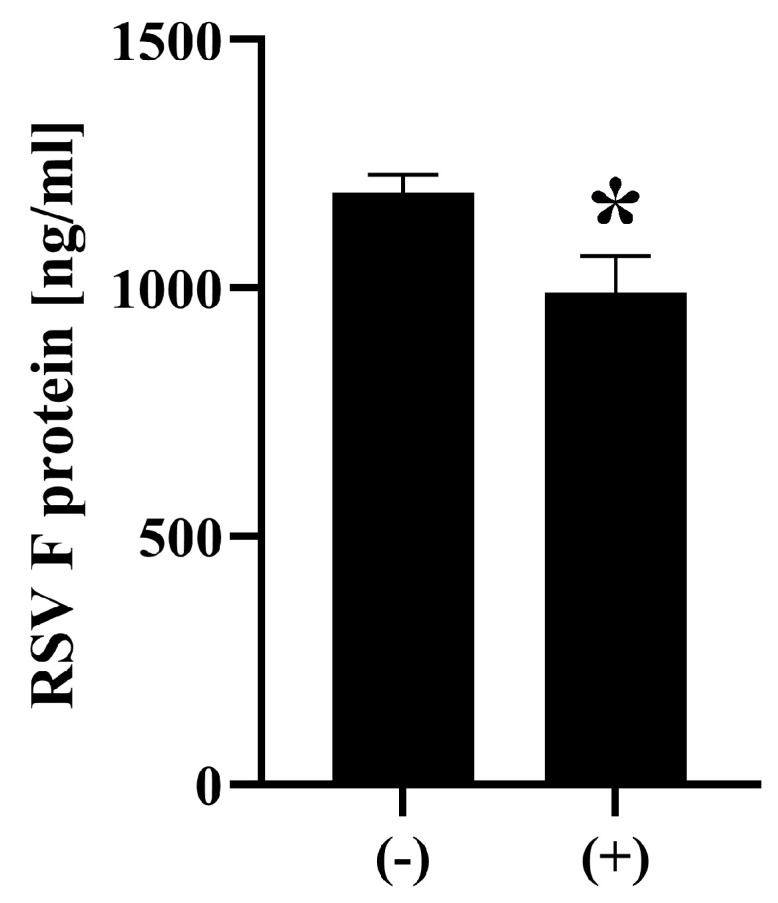
Decrease in the levels of intact RSV F protein following ozone treatment. RSV (VR-1540) samples were treated with ozone (0.5 ppm, 1 h (+)) or subjected to fan operation only (−). RSV F protein levels were quantified using an enzyme-linked immunosorbent assay (ELISA) targeting RSV F protein. A significant reduction in intact F protein was observed in the ozone-treated group (+) compared to control counterparts (−). Differences where *p* < 0.05 (*) versus control (−) were considered significant when verified by the Mann–Whitney U test.

Next, we investigated the effect of ozone on viral surface protein RSV F using ELISA. ELISA analysis showed that ozone-treated RSV samples (0.5 ppm, 1 h (+)) exhibited 990.5 ± 73.5 ng/mL of intact F protein, which is a statistically significant ~17% reduction compared to untreated control samples (1192.0 ± 18.2 ng/mL) (Figure 5).

The SFG1210 device displays advantages. First, the present study showed that the SFG1210 ozone generator reached the target concentration of 0.50 ppm within 160 s, indicating a relatively good build-up rate. Second, the design features of SFG1210 facilitate low susceptibility to RH, which commonly destabilizes the performance of discharge devices with a conventional configuration [31,32]. Third, the embedded electrode design promotes highly uniform micro-discharges along the dielectric surface [31,32]. By ensuring a consistent electrode–dielectric interface and a controlled discharge gap, SFG1210 achieves spatially homogeneous plasma generation. Fourth, the durability of the device is enhanced by the absence of direct electrode exposure to ozone, reducing maintenance costs and downtime [31,32]. These features of SFG1210 enhance the practical application of DBD-based ozone generation technology.

The present study demonstrated that RSV spotted onto glass coverslips is effectively inactivated within 1 h after exposure to relatively low-concentration ozone (0.5 ppm) generated by the SFG1210 device. Specifically, more than a 6-log reduction in viral infectivity was observed after 1 h exposure to ozone. The CT value of approximately 30.86 ppm·min at 1 h in the present study was sufficient for this inactivation effect, with a stable temperature of around 26 °C and RH over 70%, which are known to influence the efficacy of ozone-based disinfection [43]. Importantly, NOx remained below detectable levels throughout the treatment in this DBD system, suggesting that the observed antiviral effects are attributable to ozone alone and not to any cogenerated NOx. In addition, biochemical evaluation of ozone-treated RSV supports this conclusion. Ozone exposure led to a significant reduction in the levels of intact RSV genomic RNA, consistent with previous reports showing that ozone can induce strand breaks and base modifications in viral RNA genomes of other viruses [44,45,46].

Furthermore, in this study, a significant decrease in intact RSV F protein levels was observed following exposure of the virus to ozone. This observation suggests that ozone treatment also impacted RSV surface structures, likely through oxidation-induced conformational changes or degradation, which may contribute to the loss of infectivity [23]. In addition, another study has shown that lipid peroxidation disrupts the integrity of viral envelopes and compromises their ability to attach and fuse with host cell membranes [47]. Ozone can oxidize amino acid residues within viral surface proteins, leading to conformational changes that render the virus non-infectious. Together, these findings indicate that ozone acts through multiple mechanisms, damaging both the viral genome and surface proteins, leading to efficient viral inactivation. The present study supports and extends previous knowledge of ozone-induced viral inactivation and demonstrates its practical potential for surface disinfection [48].

However, there are several limitations in the present study that should be acknowledged. First, this study used a single ozone concentration (0.5 ppm) with an exposure time of 1 h. Variations in factors such as ozone concentration, exposure time, CT value, temperature and RH were not examined. This work was designed as a proof-of-concept study to assess the antiviral efficacy of low-concentration ozone under realistic and safe exposure conditions. As such, further analysis is needed of the dose–response relationship to optimize the treatment parameters as well as a complete evaluation of its broader applicability. Second, only glass among many possible environmental surface types was evaluated. The possible impact of a physiological matrix (sometimes referred to as organic load or soil load, but representing blood, other bodily fluids, or dirt) on inactivation efficacy was not evaluated. In addition, the experimental setup involved applying RSV in a spot format on glass coverslips, which may not accurately reflect real-world conditions such as virus-contaminated surfaces encountered in pediatric healthcare and public settings (e.g., plastic, stainless steel, and fabric), which differ in porosity, and surface energy, leading to differences in ozone reactivity. Therefore, the observed inactivation efficacy using a glass surface might not be directly applicable to all environmental conditions [48]. Third, a laboratory strain of RSV (VR-1540) was used in this study, and the effectiveness of ozone treatment against clinical isolates or other subtypes of RSV as well as other viruses such as different enveloped viruses and non-enveloped viruses remains to be tested [49]. The high susceptibility of enveloped viruses to ozone observed in the present study may be attributable to the oxidative vulnerability of their lipid membranes and viral surface proteins, as hypothesized in previous reports [16,17]. Non-enveloped viruses, which possess robust capsid structures, are hypothesized to show greater resistance to ozone-mediated inactivation [50]. This structural difference would be a critical consideration in the design of effective ozone-based disinfection protocols and should be further considered. Fourth, the present study demonstrated that our SFG1210 ozone generator achieves a relatively good performance at the target concentration of ozone (0.50 ppm within 160 s). However, quantitative benchmarking of different ozone generating devices through head-to-head testing using identical chamber volumes will be essential to validate performance.

Nonetheless, the findings of this study provide important data for ozone-based inactivation methods of RSV. Indeed, this represents an important step towards the safe disinfection of surfaces in pediatric healthcare and public settings, including hospitals, early childhood education, and pediatric daycare centers. Previous studies have shown the potential of ozone to control respiratory viruses under high-humidity conditions [16,17], while our results reinforce understanding of the mechanisms of ozone-induced RNA and protein disruption. Future research should expand to include a wider range of enveloped and non-enveloped viruses. Such studies will establish a broader evidence base for ozone-based virus inactivation.

In conclusion, the SFG1210 ozone generator is a promising tool for maintaining hygiene standards against RSV. Furthermore, the present study suggests that internal and external components of RSV can be affected by ozone, indicating that the virucidal mechanism of ozone may involve direct oxidative modification of viral surface proteins and nucleic acids. In addition, these findings suggest the potential real-world applicability of ozone disinfection under low-concentration, non-contact conditions in healthcare and childcare environments. The methodology developed in this study represents a safe and effective strategy for infection control without any associated chemical residues. However, to date, only a limited number of surface materials and virus strains under limited additive conditions have been analyzed. Further examination of the interaction between ozone and RSV on different material surfaces using various RSV strains and clinical isolates in the presence of other additives (e.g., proteins, including physiological substrates, salts, or other organic loads) are necessary prior to the practical application of this methodology.

## Data Availability

The original contributions presented in this study are included in the article/Supplementary Materials. Further inquiries can be directed to the corresponding author.

## Supplementary Materials

The following supporting information can be downloaded at https://www.mdpi.com/article/10.3390/microorganisms13112611/s1. Figure S1. Appearance of RSV spots after 24 h ozone treatment. RSV samples were spotted onto glass coverslips and exposed to either 0.5 ppm ozone gas for 24 h (+) using an ozone generator (SFG1210K-F) or to fan operation only (−). The untreated spots (−) maintained a red appearance, whereas the ozone-treated spots (+) turned white, indicating discoloration. Figure S2. Appearance of RSV spots after 1 h ozone treatment. RSV samples were spotted onto glass coverslips and exposed to either 0.5 ppm ozone gas for 1 h (+) using an ozone generator (SFG1210K-F) or to fan operation only (−). Both of the untreated spots (−) and the ozone-treated spots (+) retained the red color.
