# Supplementary material for: Inactivation of Respiratory Syncytial Virus by Ozone Generated via Dielectric Barrier Discharge Technology with Decrease in Intact Viral Surface Protein
Source: Microorganisms. 2025 Nov 16;13(11):2611. doi: 10.3390/microorganisms13112611 (PMC12654626; doi:10.3390/microorganisms13112611)
Supplement: Supplementary file 1 [file microorganisms-13-02611-s001.zip › microorganisms-3934958-supplementary.pdf]

Supplementary Materials

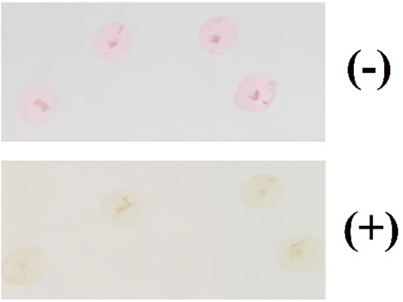

Supplementary Figure S1. Appearance of RSV spots after 24 h ozone treatment. RSV samples were spotted onto glass coverslips and exposed to either 0.5 ppm ozone gas for 24 h (+) using an ozone generator (SFG1210K-F) or to fan operation only (-). The untreated spots (-) maintained a red appearance, whereas the ozone-treated spots (+) turned white, indicating discoloration.

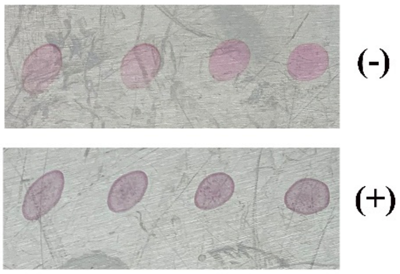

Supplementary Figure S2. Appearance of RSV spots after 1 h ozone treatment. RSV samples were spotted onto glass coverslips and exposed to either 0.5 ppm ozone gas for 1 h (+) using an ozone generator (SFG1210K-F) or to fan operation only (-). Both of the untreated spots (-) and the ozone-treated spots (+) retained the red color.
